# Forecasting and modeling of the COVID-19 pandemic in the USA with a timed intervention model

**DOI:** 10.1038/s41598-022-07487-8

**Published:** 2022-03-14

**Authors:** Gary D. Hachtel, John D. Stack, Jordan A. Hachtel

**Affiliations:** 1 Department of Electrical, Computer, and Energy Engineering, University of Colorado, Boulder, CO 80309 USA; 2 Department of Physics, University of Illinois, Urbana, IL 61801 USA; 3 Oak Ridge National Laboratory, Center for Nanophase Materials Sciences, Oak Ridge, TN 37831 USA

**Keywords:** Diseases, Mathematics and computing

## Abstract

We propose a novel *Timed Intervention* *S*, *P*, *E*, *I*, *Q*, *R*, *D* model for projecting the possible futures of the COVID-19 pandemic in the USA. The proposed model introduces a series of timed interventions that can account for the influence of real time changes in government policy and social norms. We consider three separate types of interventions: (i) *Protective interventions*: Where population moves from susceptible to protected corresponding to mask mandates, stay-at-home orders and/or social distancing. (ii) *Release interventions*: Where population moves from protected to susceptible corresponding to social distancing mandates and practices being lifted by policy or pandemic fatigue. (iii) *Vaccination interventions*: Where population moves from susceptible, protected, and exposed to recovered (meaning immune) corresponding to the mass immunization of the U.S. Population. By treating the pandemic with timed interventions, we are able to model the pandemic extremely effectively, as well as directly predicting the course of the pandemic under differing sets of intervention schedules. We show that without prompt effective protective/vaccination interventions the pandemic will be extended significantly and result in many millions of deaths in the U.S.

## Introduction

The pandemic has led to increased world interest in epidemiological modeling and prediction research, much of which focuses on extensions of the dynamic SIR model developed for generalized modeling of pandemics and applied to the spread of the plague on the island of Bombay between 1905 and 1906^1^. Some of the recent relevant academic research has been reported in References^2–5^. Here we propose a new model which can, by parameter assignment, be reduced to the model of^2^, which appeared in February, 2020. Like the model of Reference^2^, the model proposed here partitions the total population into 7 states (or “compartments”). Unlike the model of^5^, no explicit delays are included in the formulation. Rather, delays arise as an inherent property of the parameter assignment, and the structure of the state equations in Eq. (1) below.

The growth of populations as diverse as yeast cultures and national populations was studied in a very general setting in^6^. In that work, Pearl showed that the logistic function provided a quantitative model for the growth of various populations. Similarly, Rappole^7^ used logistic functions to model habitat control of avian populations, again employing logistic modeling. In our study, it is found that logistic-like functions model sink states like the recovered and deceased states *R* and *D*, but that Gaussian-like functions are needed to model the non-sink states exposed, infected, and quarantined states *E*, *I*, and *Q*, since these states all exhibit extrema in their time evolution. The timed interventions directly control the waveforms of the susceptible and protected states *S*, and *P*, which would normally be modeled by logistic-like functions but have a piecewise waveform in our model that is only logistic-like between successive interventions.

Epidemics are not rare but are frequent visitors to the world stage, like earthquakes and category 5 hurricanes. As a result, there is an inherent need for established and validated long term forecasting of epidemics, to help guide policy making decisions and establish cost-benefit analyses^8,9^. This is all the more critical during the current COVID-19 crisis.

## Results

### The COVID-19 pandemic in the USA

**Figure 1:**
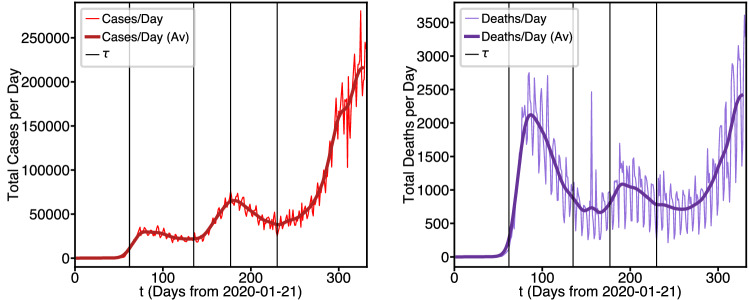
Smoothed cases per day and deaths per day from source data exhibiting inflection points.

Figure 1 demonstrates the primary motivation for developing the proposed timed intervention model.

Here, we consider the COVID data from the U.S. as a whole from the date of the first recorded case (January 21st, 2020) to the time of submission of this manuscript (December 16th, 2020). The smoothed (7-day rolling average) waveform appears to have 5 distinct phases. The first, for $0< t \le \ 62$, could be called a “preliminary spread” phase. In this phase the outbreak established itself in the state of Washington. However, we see that around day 64 the preliminary spread began to slow, reaching a peak in cases per day at around day 78 and then beginning to decrease. Day 62 would be March 23rd, 2020 which was approximately the time when stay-at-home orders were beginning to be issued nationwide. Thus, from $62< t \le \ 140$, the number of new cases per day stabilizes and starts to decrease resulting in a significant decrease in deaths per day. We call this a “protective” phase. However, we see that at around day 140 the cases begin to trend sharply upwards again, which likely corresponds to the lifting of the stay-at-home orders and the general population relaxing their commitment to social distancing guidelines, we call this a “release” phase. It can be seen clearly that the release phase lasts from $140< t \le \ 185$, where another protective phase begins which goes from $185< t \le \ 230$. The most alarming trend is that since day 230 the U.S. has been in an extended release phase, with cases per day and deaths per day spiking to unprecedented levels. In this data the influence of four effective interventions can be observed, along with a clear need for a fifth protective intervention. It is important to note, that the pandemic will, of course, have progressed beyond these dates by the time of publication, so our results here are designed to exemplify how one can use the timed intervention model to make predictions according to these data specifically. We also provide the code and an interactive iPython notebook (see “Methods”) so that researchers can extend the modeling and predictions to future data.

The classical predator-prey models of the Lotka-Volterra equations that first appeared in^1^ do not show such articulated multi-phase behavior. Similarly, more modern work on pandemic modeling^2^ also does not show such behavior, necessitating the development of a new model.

### Proposed timed intervention model

Our dynamical pandemic model is represented by the following state equations

1 $$\begin{aligned} \begin{aligned} {\dot{P}}&= \alpha (t) S - \phi (t) P = { Protected\ Population} \\ {\dot{S}}&= - \beta \frac{IS}{{\mathbf {N}}} - \alpha (t) S + \phi (t) P= { Susceptible\ Population}\\ {\dot{E}}&= \beta \frac{IS}{{\mathbf {N}}} - \gamma E ={ Exposed \ Population}\\ {\dot{I}}&= \gamma E -( \delta + \rho + \nu ) I = { Infected \ Population}\\ {\dot{Q}}&= \delta I - (\lambda + \kappa )Q = { Quarantine/Isolated\ Population}\\ {\dot{R}}&= \lambda Q + \nu I = { Recovered\ Population}\\ {\dot{D}}&= \kappa Q + \rho I = { Deceased\ Population}\\ \end{aligned} \end{aligned}$$

**Figure 2:**
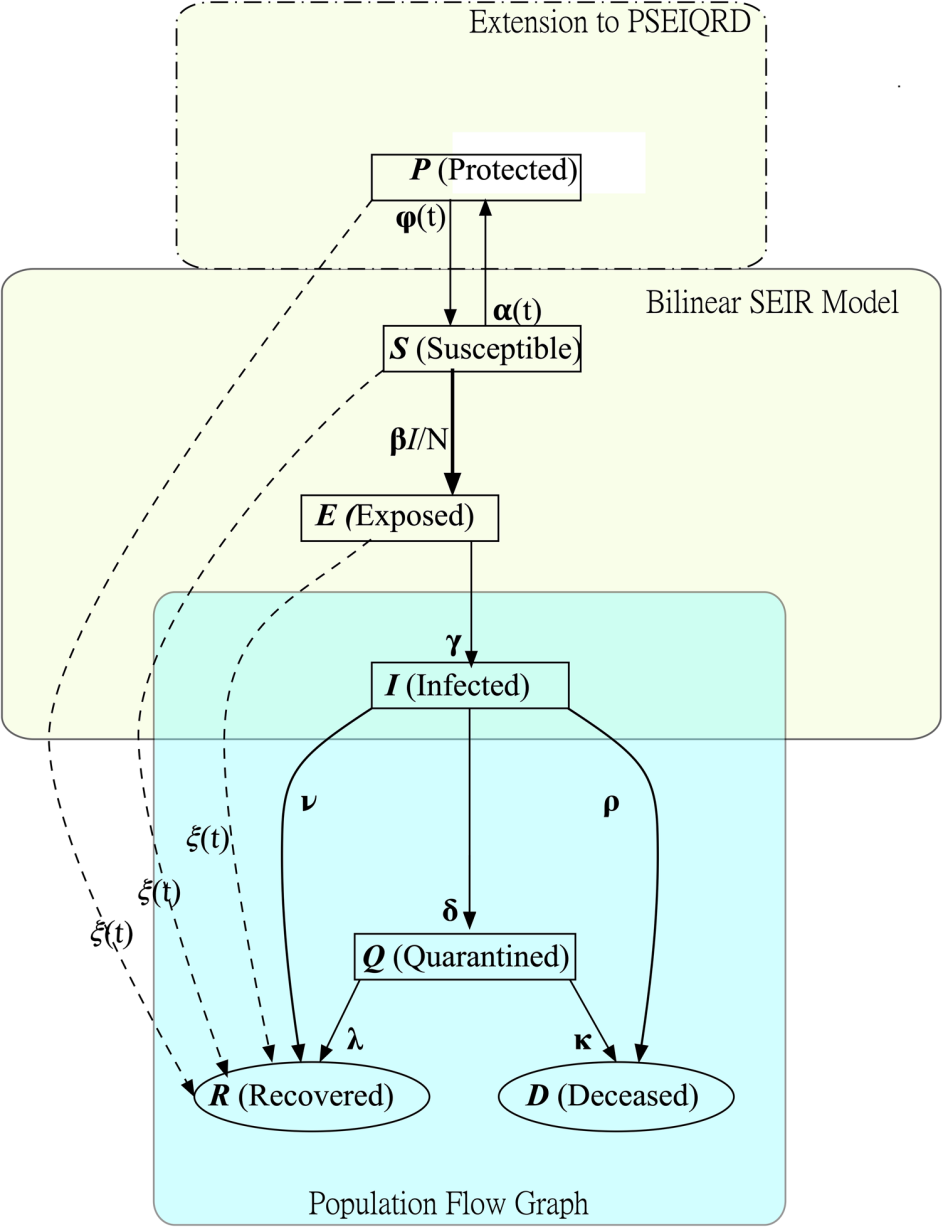
State transition graph of the model.

Here the seven state variables include: *Protected, (P)* corresponding to the population that is not likely to be infected due to following stay-at-home orders and social distancing guidelines, *Susceptible, (S)* the population that can potentially be exposed and infected, *Exposed, (E)* the population who have come in close contact with an infected person, *Infected (I)* the population who have contracted COVID-19, *Quarantined, (Q)* the population who have been isolated from the general population after infection (*not* exposure) to prevent further spread of the disease, *Recovered, (R)* the population who has recovered from COVID-19 and is now (for the most part) immune, *Deceased, (D)* the population that has died from COVID-19. Also, here we introduce the bilinear term, $B=-\beta \frac{IS}{N}$. *B* is of the utmost importance for our model, because the magnitude and timing of its maxima dictates the magnitude and timing of the maxima in the all the populations and hence controls the course of the pandemic. It should be mentioned at the outset that state *P* of this model does not strictly correspond to the state of the same name in^2^, where *P* stood for an auto-immune population. In the present model, *P* stands for a protected, i.e. “sheltered in place” or “social distancing”, population and any immune population is lumped into the *R* state. A graph showing the directional flow of population is given in the state transition diagram of Fig. 2. Increments of population flow systematically through this graph like clockwork, in which the time step is 1 day.

The lack of a *E* to *S* arrow in this figure indicates that, once exposed, members of the exposed population cannot rejoin the ranks of the susceptible population. Similarly, once infected, members of the infected population cannot rejoin the members of the exposed population. This implies that individuals *cannot be re-infected*. The only feedback loop is from *S* to *P* and back. Note that the long dashed arrows only exist in the presence of vaccination, as discussed below.

This model is *conservative*, in the sense that it assures the total population is constant. That is,

2 $$\begin{aligned} \begin{aligned} {\dot{S}} + {\dot{P}} + {\dot{E}} + {\dot{I}} + {\dot{Q}} + {\dot{R}} + {\dot{D}}&= 0,\\ S + P + E + I + Q + R + D&= {\mathbf {N}} , \\ S(t), P(t) , E(t) , I(t) , Q(t) , R(t), D(t)&\ge 0,\ \forall \ t. \\ S(t), P(t) , E(t) , I(t) , Q(t) , R(t), D(t)&\le {\mathbf {N}},\ \forall \ t. \end{aligned} \end{aligned}$$

### The timed interventions of the model

We define $B= \beta \frac{I S}{{\mathbf {N}}}$ as the bilinear term of Eq. (1). The quasi-stable dynamical system defined by these equations must eventually reach *equilibrium* in which ${\dot{S}}\longrightarrow 0$ and $S(t)\longrightarrow S_F$, and similarly for *P*, *E*, *I*, *Q*, *R* and *D*. Thus we can succinctly state definitions for 2 types of timed interventions at time $t =\tau$:

- Protection–if $\alpha S(\tau ) > \phi P(\tau )$, then ${\dot{P}}(\tau ) > 0$ and population flows from *S* to *P*;
- Release–if $\alpha S(\tau ) < \phi P(\tau )$, then ${\dot{P}}(\tau ) < 0$ and population flows to *S* from *P*;

Note that intervention types are defined by the state of the system at the time of intervention, that is, at the start of the associated interval. Although it often happens that these conditions persist throughout the interval, sometimes they do not, due to the influence of the bilinear term *B*. The role played by *B* is crucial, and shall be closely monitored here.

The primary objective of our research is to define, seek, and realize (theoretical) *virus extinction*–Suppose there exists $\tau _{\Omega }$ such that $\forall t >\tau _{\Omega }$, $\alpha S_F = \phi P_F = E_F = I_F=Q_F =0$, but $R_F$ and $D_F$ are positive constants. In this case, since $E_F=I_F=Q_F = 0$ and $R_F>0$, the virus, but not the host, is extinguished, and the pandemic is over. Model parameter values have been shown to exist which demonstrate the existence of viral extinction, without the entire population being infected and transitioning into the R or D end states.

Fortunately the condition that $E_F = I_F=Q_F = 0$ is not a rare case but is a guaranteed property of any equilibrium state of the model, as we now show. Here we use the definition $B=\beta (IS)/{\mathbf {N}}\doteq bIS$, and define $\epsilon = \alpha S - \phi P$.

#### **Theorem 1**

*Consider any set of inputs for which the dynamical system of Eq.* (1) *reaches an equilibrium state* $S_F,P_F,E_F. I_F,Q_F,R_F,D_F$*. Then* *(i)* $B_F = E_F = I_F=Q_F = 0$*, (ii)* $\alpha S_F = \phi P_F > 0$*, (iii)* *E,* *I, and* *Q* *in general exhibit at least one local maximum, and (iv)* *R* *and* *D* *exhibit logistic waveforms.*

#### *Proof*

By Eq. (1) and the above definitions, ${\dot{S}}=-B -{\dot{P}} =-(B + \epsilon )$, where $\epsilon \doteq \alpha S - \phi P$ and similarly ${\dot{E}} = B - \gamma E$. So when equilibrium is reached, (denoted by *F* subscripts) we have ${\dot{E}} = 0$, so $\gamma E_F = B_F = (\beta /N) I_F S_F$. In equilibrium ${\dot{I}}={\dot{P}}=0$ as well, so $\gamma E_F=(\delta + \rho + \nu )I_F$ and similarly $\alpha S_F = \phi P_F$ and $\epsilon _F=0$. Also, in equilibrium ${\dot{S}} + {\dot{E}} = 0$, so $\gamma E_F = -\epsilon _F = 0$ and thus $E_F=0$, $B_F=0$, and $I_F=0.$ Finally ${\dot{Q}} = 0$ implies $Q_F =(\delta I_F )(\lambda +\kappa ) =0$. Since *E*, *I* and *Q* start non-negative and asymptotically approach 0 for sufficiently large *t*, their shapes must exhibit at least one local maximum (possibly at $t=0$, resulting in exponential decay). Finally, since ${\dot{R}}=\nu I + \lambda Q$, *R* is the integral of a Gaussian-like peak and is hence logistic (i.e. a s-shaped step-like waveform). The same holds true for *D*. In the exponential decay case, the sink populations (*R* and *D*) increase asymptotically towards their final values. $\square$

#### **Lemma 1**

*Define* $\mu _B$ *to be the time of the final maximum of* *B*(*t*) *and similarly for* $\mu _E$*,* $\mu _I$*, an* $\mu _Q$. *Then* $\mu _B< \mu _E< \mu _I <\mu _Q.$

#### *Proof*

Because in one day only a fraction of the population in a given state transfers to the other states, the complement of this fraction remains in the state (see Fig. 2), the increase of *E* in the $i_{th}$ time step which brought *E* to its maximum, cannot be propagated to *I* until at least the $(i+1)_{th}$ and possibly much later. The same argument applies to *B* and *Q*, *mutatis mutandis*. $\square$

#### **Theorem 2**

*For any set of transition coefficients,* $\beta ,\gamma ,\delta ,\nu ,\rho ,\lambda ,\kappa$*, there exists a set of of intervention coefficients* $\alpha (t),\phi (t)$ *that lead to virus extinction.*

#### *Proof*

Consider the function *B*(*t*) at time $t = \mu _E$ (the final local maximum of *E*). Since ${\dot{E}}(\mu _E) = 0$, we have $B(\mu _E) = \gamma E(\mu _E)$ and since by the Lemma $\mu _B < \mu _E$, it follows that $B(\mu _E)<B(\mu _B)$, and since there are no further maxima of *B* or *E*, it follows that both decrease to 0 asymptotically for $t>\mu _E$. Next consider the interval $(\mu _E < t \le \mu _I)$. In this interval, *I* is increasing, but for sufficiently large values of the ratio $\phi (t)/\alpha (t)$, *P*(*t*) is increasing. But for $t>\mu _Q$, *I* is strictly decreasing and at equilibrium $P_F>0$ and due to the conservative property, $D_F<N$. Thus, the virus, but not the host, is extinguished. $\square$

### Interventions and COVID-19 in the USA today

The real world per day waveforms of Fig. 1 are not Gaussian or logistic but appear to be piecewise combinations of such waveforms. Although there have been no formal governmental nationwide interventions, the data behaves as if there had been. In our experience, the pandemic appears to be lurching through a series of *punctuated equilibria*. Here we attempt to model these auto-interventions as closely as possible.

Note that in Fig. 1, vertical lines have been drawn at 64th, 150th, 178th and 250th days. These are the days which we have found that changes in $\alpha$ and $\phi$ can generate the best match to data. The model attempts to capture these changes by defining the time dependence of the coefficients $\alpha (t)$ and $\phi (t)$ of Eq. (1) to be piecewise constant. That is, the overall interval [0, *T*] is partitioned into $n+1$ subintervals $i = 0,1,\ldots ,n$ with $T\equiv \tau _{n+1}$.

3 $$\begin{aligned} \begin{aligned}{}&\alpha (t)\ = \alpha _i, \ \phi (t) = \phi _i, \tau _i \le t< \tau _{i+1}\\&\alpha (t)\ = \alpha _n, \ \phi (t) = \phi _n, \tau _n \le t < \tau _{n+1}=T\\ \end{aligned} \end{aligned}$$

To understand $\alpha$ and the other population transition rate parameters, suppose that some time *t*, $S(t)= 3\times 10^8$. If $\alpha = 0.2$, then at time $t+\Delta = t + 1$, if *B* and *P* happen to be negligible, $S(t+1) = S(t)-\alpha \cdot S(t)\ or\ (3\times 10^8-0.45\cdot 3\times 10^8) =1.35\times 10^8$. In this case 135 million people are moved from *S* to *P* in a single (unit) time step of 1 day.

### Assignable parameters

The model definition is completed by the specification of the following assignable parameters:

- a 9-vector *x* of assignable transition rate coefficients,where
  $$\begin{aligned} x =\alpha _0,\phi _0,\beta ,\gamma ,\delta ,\lambda ,\kappa ,\nu ,\rho \end{aligned}$$
  (see the state transition dependency graph of Fig. 2);
- *n* intervention time parameters $\tau _i,\ i = 1,2,\ldots ,n$;
- 2*n* assignable intervention rate parameters $\alpha _i$ and $\phi _i,\ i = 1,2,\ldots ,n$.

Note that the initial rate parameters $\alpha _0$ and $\phi _0$ are the intervention rate parameters for the first interval. In general, there are $9+3n$ assignable parameters in all, not counting the 7 initial conditions on the state variables Each of these parameters must satisfy constraints on their allowable values shown below. The default values of these parameters (shown in square brackets) are the only set of transition parameters used to obtain the numerical results given here.

4 $$\begin{aligned}{}&\begin{aligned} 0&\le \alpha _0[0] \le 1 \equiv { initial\ S\ to\ P\ transfer\ rate }\\ 0&\le \phi _0[0.001] \le 1 \equiv { initial\ P\ to\ S\ transfer\ rate}\\ 0&\le \beta [.92] \le 1 \equiv { infection\ rate} \\ 0&\le \gamma [.0305] \le 1 \equiv { rate\ of\ infections\ per\ exposed}\\ 0&\le \delta [.02] \le 1 \equiv { rate\ of\ quarantines\ per\ infected} \\ 0&\le \lambda [.07] \le 1 \equiv { quarantined\ recovery\ rate}\\ 0&\le \kappa [\lambda /19] \le 1 \equiv { quarantined\ mortality\ rate}\\ 0&\le \nu [.15] \le 1\equiv { unquarantined\ mortality\ rate}\\ 0&\le \rho [\nu /20] \le 1\equiv { unquarantined\ recovery\ rate}\\ \end{aligned}\end{aligned}$$

5 $$\begin{aligned}{}&\begin{aligned} 0&\le \alpha _i \le 1 \equiv { protection\ rate\ of\ intervention\ } i, i = 1,\ldots ,n\\ 0&\le \phi _i \le 1 \equiv { release\ rate\ of\ intervention\ } i = 1,\ldots ,n\\ \end{aligned} \end{aligned}$$

These define the constraints on the values the components of the *n*-vectors $\alpha$ and $\phi$. A parameter set $p\in \tau \times x \times \alpha \times \phi$ which satisfies the above constraints is called *feasible* and the solution of Eq. (1) corresponding to such a *p* is called a *future* of the pandemic. A solution for an *infeasible* parameter set is a *behavior of the model* but not a *future of the pandemic.*

For the purpose of understanding the delays observed in the evolution of the pandemic, consider the node for the infected population *I*(*t*) in Fig. 2. There are 3 possible state transitions out of this state, in which members of the population *I* are either:

- quarantined with rate $\delta$;
- recovered with rate $\nu$;
- deceased with rate $\rho$;

Of course some fraction of the infected population *I* do not transfer out but remain in *I*. Delays arise because it may take multiple, even many, days for increments of *I* arriving from *E* at any given time step to propagate forward. This argument applies to the non-terminal states *S*, *P*, *E*, and *Q* as well. Numerical studies require the choice of the 7 initial conditions and the $9 + 3n$ free parameters.

After first reviewing prior work on pandemic modeling, we shall compare the deaths and deaths per day data to date to the prediction of the model for the given rate coefficients and intervention parameters.

### Comparison to prior published work on pandemic modeling

As stated above, our proposed model is an extension of that proposed by Peng et al.^2^, which not only differs in the definitions of ${\dot{S}}$ and ${\dot{P}}$, and of *I*, ${\dot{R}}$, and ${\dot{D}}$, but also includes the *n* timed interventions with their associated 3*n* rate constants as well. Conceptually, our protected state *P* represents the isolated, or “sheltered in place” population rather than the innately immune. These differences enable the model to obtain excellent qualitative and quantitative results for the USA as a whole when compared to real world data to date. More recently, a comparable SEIR model was presented by Adak et al.^10^, who showed that their solutions existed, were unique, and bounded. They treated both viral extinction and endemic cases, and offered theorems comparable to our Theorem 1. They did not include timed interventions or vaccination. They gave results for the cumulative Confirm Cases Death Counts for the Covid-19 epidemic in Spain from February to October 1920. Over that period the shape of their death curves were of course similar to ours for the United States. Another interesting alternative and comparable approach is given by Liu et al.^11^, which introduced an asymptomatic state in the SEIR extension and used neural networks to solve the parameter identification problem.

Equations (3) and (4) define $9+3n$ free parameters. The parameter space is $9+3n$-dimensional (or $16+3n$ if we include the initial conditions for the 7-dimensional state space). The essence of modeling is to solve the *parameter identification problem*: Find the point in the $9+2n$ dimensional parameter space for which the model results best fit real world data. To solve this rigorously, a second round of research is proposed: find the optimal $2n+9$ ($2n+16$ ) parameter assignments using the approach of Reference^12^, starting from the quasi-optimal parameter set used throughout this paper. Equation (1) specifies a set of 7 ordinary differential equations in which the ${\dot{E}}$ and ${\dot{S}}$ equations have a *bilinear* form which first appeared in Equation 29 of Reference^1^. They were also used by Anderson and May^13^ to model a wide variety of infectious diseases. Such equations can exhibit unstable, and even chaotic, behavior as was first shown by Lorenz for modeling of atmospheric convection^14^, and has also been observed in the modeling and analysis of viral epidemics^15^. Oscillatory solutions arise in practice for extreme values of $9+2n$ parameters, but the these can be avoided by judicious values of the upper and lower bounds of Eq. (2). In our limited exploration of the multi-dimensional parameter space, no chaotic behavior has been observed. Perhaps the last 4 linear equations constitute a dissipative damping effect which inhibits the oscillatory behavior which might be generated by the state equations for *S* and *E*.

Early epidemiological research^1^ focused on detailed qualitative reasoning and Taylor series expansions. The lack of computing facilities did not stop them from considering a formulation even more general than that discussed here. They derived the SIR equations as a special case, and they found a special case solution in terms of hyperbolic functions for $z = R+D$. In this solution ${\dot{z}}$ appeared to be Gaussian and *z* therefore a logistic function , and they were able to assess viral extinction in their analysis. Similar results from the same era were obtained in^6^ whose author emphasized the same classification of population growth functions as logistic and Gaussian (except in his Fig. 8 he used the term “first derivative of logistic”). His solutions were consistent with Theorem 1 although no theorem was stated. Many more contemporary results, for example^7^ and^5^ had their own extensions to the SEIR model but the results could be similarly categorized. None of these extended SEIR models achieved strong matches to COVID-19 data to date, because they were limited to one rise and fall of the bilinear term *B*(*t*). With this limitation, the pandemic rises too rapidly, and then falls to extinction too rapidly to match data to date.

Statistical models^16,17^ have achieved good short term matches, but do not consider extinction, which is a long term event. Such models cannot effectively forecast the duration of the pandemic. Recently, modeling papers have appeared that are sufficiently general to accommodate the influence of social and politcial changes in the midst of the pandemic^4^ or focus on Health Care requirements and localized results^18^. Reference^3^ is worthy of note since they prove that any accurate analysis of the pandemic must be an extended SEIR model. Our work, also an extended SEIR model, extends it further by using the timed interventions. The necessity to treat the pandemic through multiple distinct epochs, in which the bilinear term rises and falls as is clearly evidenced in the data analyzed. The timed-intervention SPEIQRD model permits direct projections of mortality and pandemic extinction, which is were clearly needed during the COVID-19 pandemic.

Further research was stimulated by the explosive growth of Covid-19. Cao and coauthors^19^ offer a neural network modeling solution and included an extensive survey of the contemporaneous literature. Atkeson^20^ gave an economist’s perspective on SEIR epidemiological models. In^21^ the “accuracy” of short term predictive models are compared.

### Extending data fitting results into the future with the timed interventions model

**Figure 3:**
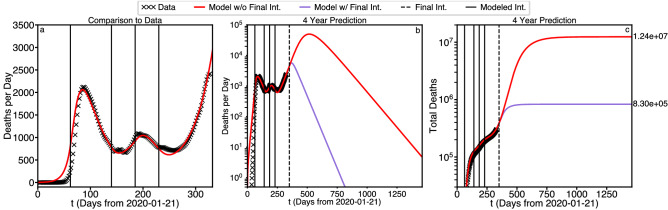
Best fit to deaths per day and total deaths, with and without a final intervention.

Figure 3 illustrates the ability to use timed interventions to both tune the model to real data as well as perform long term predictions. In Fig. 3a we show the match between the model with timed interventions at $\tau _i=62, 140, 185, 230$ as compared to data, the corresponding values for $\alpha _i$ and $\phi _i$ are shown in Table 1. By changing the flow of population between S and P we achieve an extremely good match to data extending all the way through the most recent date considered (Dec. 16th 2020), validating the effectiveness of our model.

**Table 1:** Values of best fit timed intervention parameters for optimum match to data.

$\tau _i$	$\alpha _i$	$\phi _i$
0	0.0	0.001
62	0.148	0.004
140	0.097	0.031
185	0.085	0.003
230	0.029	0.013
350	0.085	0.003

We can then use the model to assess the influence of a potential fifth or final intervention at a date in the future. Figure 3b,c show the four-year predictions for Deaths per Day (b) and Total Deaths (c), where the modeled interventions from Fig. 3a are shown in solid lines with the new potential final intervention shown as a dashed line. Model parameter are shown in Table 1. Critically, we note that with a final intervention at day 350 (of comparable parameters to previous interventions) the deaths per day (purple curve) dips under 1 death per day in 784 days, an indication of viral extinction (more on this later). In contrast, without the final intervention (red curve) deaths per day doesn’t dip under 1 until the 1606st day, more than 4 years out. Moreover, we can see the influence these two scenarios have on the total deaths that will occur during the pandemic. The scenario without a final intervention results in 17 times more deaths than with the final intervention, meaning that prompt action could (theoretically) save more than 12 million lives.

A key focal point of this study has been forecasting the duration of the pandemic in the absence of a vaccine. Theorem 1 states that the waveforms for the size of exposed population *E*, infected population *I*, and quarantined population *Q* must asymptotically approach 0 as the duration of the simulation approaches infinity. Since the initial conditions for the size of these populations are positive, it follows that each of these sizes must reach a maximum value and then eventually decay to zero. The duration of the pandemic is, therefore, defined by how long It takes for these eventualities to occur.

Since, by definition, ${\dot{D}} = \rho I + \kappa Q$ deaths per day must behave similarly. In fact, the red curve in Fig. 3b, beyond the 5th intervention, resembles a Gaussian function.

**Figure 4:**
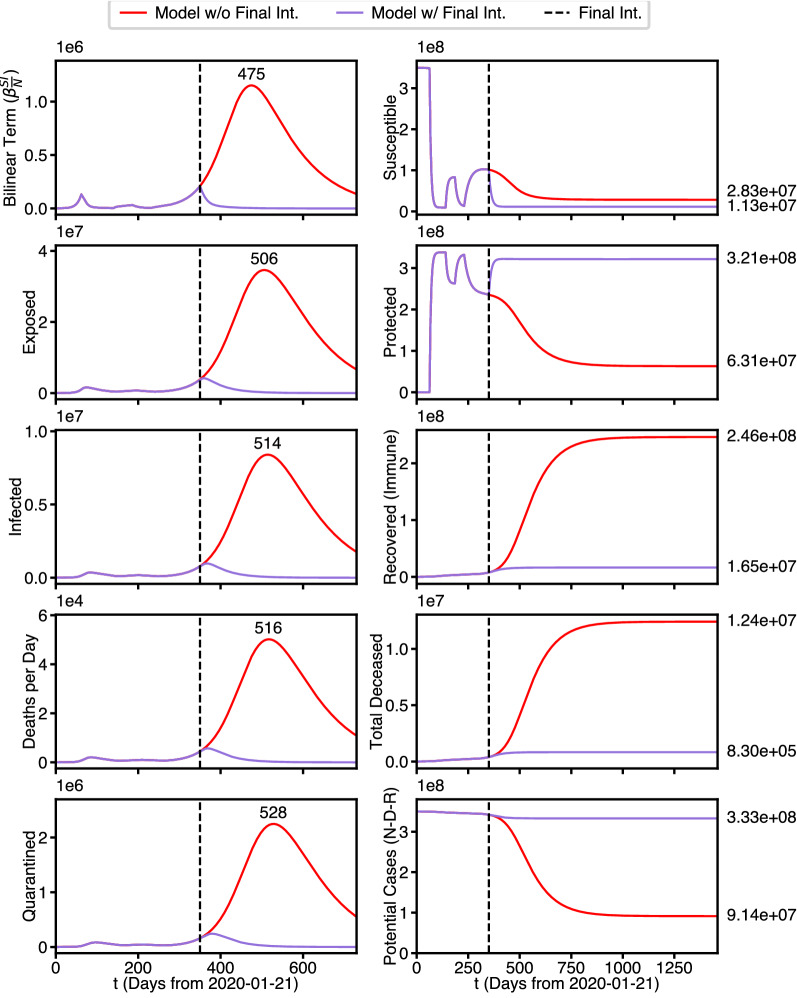
Four year predicted state population waveforms. Best fit parameters with and without final intervention.

These eventualities are illustrated by the 2 year simulation of Fig. 4. The red curves in the first column again indicate the absence of the 5th intervention. The purple curves illustrate the effect of the 5th intervention and are seen to be significantly flattened by the this intervention. The days on which the red curves reach their maximum value are shown just above these maxima, which are ordered in accordance with Lemma 1.

On the right, it is seen that in both scenarios *S* is drained by the last intervention (here extended out to a four year prediction). However, the red curve drains far less rapidly than the purple curve does in response to the strong protective 5th intervention. However the purple curve for *P* rises sharply in response to the 5th intervention, whereas the red curve drains at about the same rate as *S*. The asymptotic limit of *P*(*t*) for the purple curve reaches above $3\times 10^8$, shortly after the date of the 5th intervention. In contrast, the asymptotic limit of the red curve is nearly an order of magnitude less ($6\times 10^7$), and has not reached its asymptote even 2 years out. Note also that in the interval just before the intervention ${\dot{P}}$ and ${\dot{S}}$ tend to approach 0 and according to Theorem 1 , $\alpha S_F = \phi P_F$ at the end of that interval. We have called this effect “punctuated equilibria.”

Similarly the sink states *R* (labeled here as Recovered/Immune) and *D* approach constant values and display skewed logistic waveforms In accordance with Theorem 1. Again the gap between final death toll between the red and purple curves is approximately $1.3\times 10^7$ ( more than 13 million lives saved). Finally the bottom plot on the right illustrates $N-D$, a simple first definition of those that survive the Pandemic.

### The timing of the final intervention

As stated above, we have used theoretical interventions to determine a “best fit to data” model to simulate the behavior of the pandemic to date. During future prediction, however, we can employ future interventions, to maximize the number of survivors. These future interventions must be realizable in the political landscape of the United States. For example, in the “best case” future of Section 4 the critical last intervention increased the protected population to over 92% of the total U.S. population, and so is probably not politically feasible.

**Figure 5:**
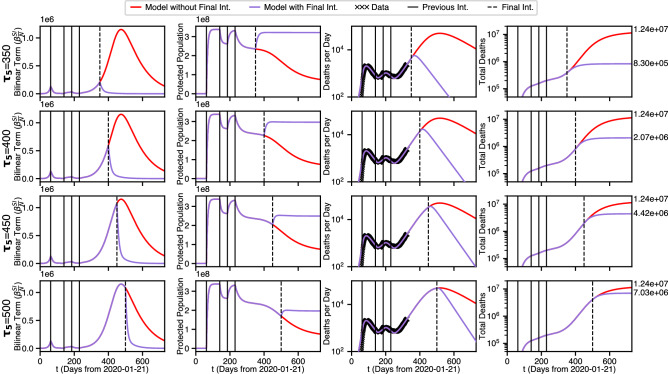
Four possible final intervention time scenarios.

We now look at some alternative futures based on variations of the timing of the final intervention, shown in Fig. 5.

This is a complex figure but it can understood as follows. Each of the 16 plots has a red curve and purple curve. The red curves correspond to the absence of the final intervention, and therefore they do not change in any given column. Each of the 4 rows correspond to a specific time value for the final intervention, for $\tau _5=350, 400,450,500$, which is denoted with a dashed line. In each row the first waveform plotted is the bilinear term $B = \beta \frac{I S}{N}$. Looking down the first column, it can be seen that the maximum of *B* creeps up the red curve until the final intervention occurs and then decreases rapidly and quasi-logistically toward 0. The second column shows the effect of final intervention on the protected population *P*. It is seen that in each row the purple curve rapidly increases and stablizes quickly at a final value, but as the onset of the final intervention becomes later and later the total population that becomes protected gets lower and lower.

In the 3rd column, we plot the deaths per day for each $\tau _5$. As a result, the date of viral extinction is pushed in to the future significantly. This shows that even with extreme protective interventions viral extension is not be achieved for nearly a year, and much longer than that if the intervention is not prompt.

In the 4th column it can be seen (purple curves) that the increase in the peak of the bilinear term incurs a corresponding increase of the corresponding final death toll, rising in the sequence $8.30\times 10^5$, $2.07\times 10^6$, $4.42\times 10^6$, $7.03\times 10^6$ as the day of the 5th intervention increases in the sequence 350, 400, 450, 500. It is seen that the final death toll essentially doubles for each 50 days of delay before the 5th intervention. A similar result happens in the third column for deaths per day as well.

It is also to be noted that with the exception of deaths per day, all of the protected scenarios show waveforms that have reached (or least come close to reaching) equilibrium within two years. However, in the $\tau _5=500$ scenario deaths per day does not go below 1 until day 924, nearly two years from the current date (dates where DPD goes below 1 shown for all scenarios in Table 2 below).

**Table 2:** Viral extinction dates and total fatalities as a function of day of final protective intervention, for scenarios in Fig. 5.

$\tau _5$	$\tau _{\Omega \dot{D}}$	$\tau _{\Omega }$	$D_F$
350	784	1073	$8.30\times 10^5$
400	868	1149	$2.07\times 10^6$
450	922	1188	$4.42\times 10^6$
500	954	1204	$7.03\times 10^6$
None	1606	2207	$1.24\times 10^7$

### Vaccination interventions

To account for the rollout of vaccination, we introduce a new coefficient to our model: $\xi$. This value represents the rate at which the population is immunized as a function of time (i.e. the rate at which population moves from *S*, *P*, and *E* to the recovered/immune state *R*. We can incorporate this into the model by treating vaccination as another timed intervention, where $\xi$ is zero before the vaccine roll out. This corresponds to the following modifications of the basic model of Eq. (1).

6 $$\begin{aligned} \begin{aligned} {\dot{P}}&= \alpha (t) S - \phi (t) P - \xi (t) P = { Protected\ Population} \\ {\dot{S}}&= - \beta \frac{IS}{{\mathbf {N}}} - \alpha (t) S + \phi (t) P - \xi (t) S = { Susceptible\ Population}\\ {\dot{E}}&= \beta \frac{IS}{{\mathbf {N}}} - \gamma E - \xi (t) E={ Exposed \ Population}\\ {\dot{I}}&= \gamma E -( \delta + \rho + \nu ) I = { Infected \ Population}\\ {\dot{Q}}&= \delta I - (\lambda + \kappa )Q = { Quarantine/Isolated\ Population}\\ {\dot{R}}&= \lambda Q + \nu I + \xi (t) P + \xi (t) S + \xi (t) E = { Recovered\ Population}\\ {\dot{D}}&= \kappa Q + \rho I = { Deceased\ Population}\\ {\dot{R}}&= \lambda Q + \nu I = { Recovered\ Population}\\ {\dot{D}}&= \kappa Q + \rho I = { Deceased\ Population}\\ \end{aligned} \end{aligned}$$

Note that each subtracted term is accompanied by an equal and opposite added term, so that the model still has the conservative property of Eq. (1). The $\xi$ terms correspond to the dashed arrows in Fig. 2.

**Figure 6:**
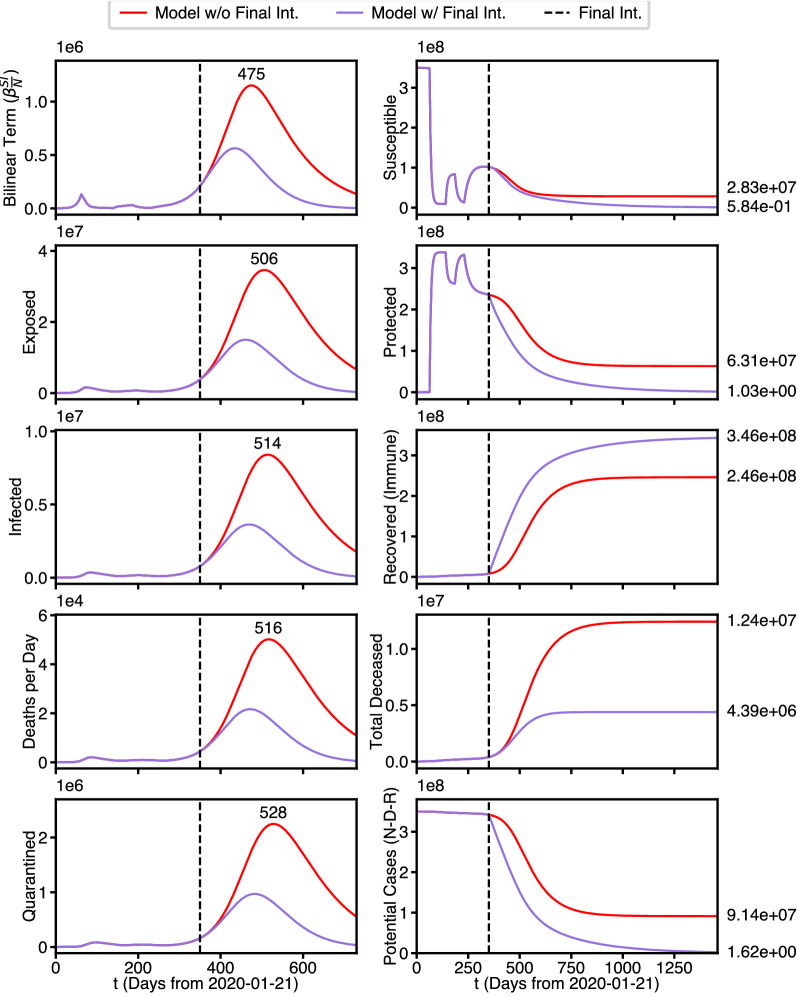
Four year predicted state population waveforms. Best fit parameters with and without a vaccination intervention.

We use the same parameters as discussed above in the intervention case but only re-use the first 4 interventions. Thus the critical final protective intervention is replaced by a vaccination intervention of severity $\xi$ that happens on the 350th day. The modified model produces the waveforms illustrated in Fig. 6.

As before, the plots on the left show waveforms for the 5 Gaussian-like waveforms for the transient populations *B*, *E*, *I*, ${\dot{D}}$, and *Q*. The red curve is identical to the red curve in Fig. 4, which corresponds to no protective interventions or vaccinations, while the purple curve now represents the vaccination intervention.

The plots on the right, however tell an entirely different story. Without vaccination (red curves) the red curves are identical to their values in Fig. 4, but with vaccination (purple curves) they now drain down to zero. Note that the third plot is now labeled Recovered (Immune), rather than Recovered. The takeaway here is that the purple curve ($3.46\times 10^8$) is substantially above the red curve ($2.46\times 10^8$), and is close to the total population.

We note that the total deaths is the case in vaccination is higher than in the case of a protective measure discussed in Fig. 4, however this is a function of rate. Here, the rate of *S* and *P* draining to *R* is much slower than the rate of *S* draining to *P* in the protective measure case, which are controlled by the parameters $\alpha$ and $\xi$ respectively. We used such values, since protective measures such as lockdowns and stay-at-home orders can instantaneously affect large number of people, while vaccines must be rolled out over a longer time scale. However, the critical benefit of vaccination can be seen by comparing the final equilibrium of Potential Cases in the three scenarios of no further intervention (red curves), protective intervention (purple curve in Fig. 4), and vaccination intervention (purple curve in Fig. 6. With no further intervention, the majority of the population contracts COVID, resulting in mass immunity and a limited number of potential future cases ($9.14\times 10^7$). For protective intervention, even though the immediate result is many saved lives, nobody becomes immune and the maximum potential cases stays near the total U.S. population ($3.33\times 10^8$) so any release interventions before viral extinction can be potentially disastrous. However, for vaccination intervention, even though more lives are lost in the short term the entire population becomes immunized resulting in the maximum potential cases dropping to zero. This is an important, possibly the most important, takeaway of our study: Whereas protective intervention can produce comparable or even superior mortality and viral extinction times, with vaccination the population becomes immune, and cannot be reinfected.

**Figure 7:**
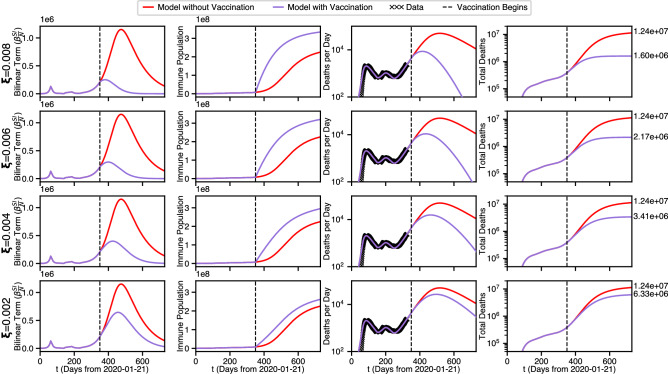
Four possible vaccination strength scenarios.

Furthermore, the mortality in the case of vaccination intervention can be improved by changing the speed of the vaccine roll-out, as shown in Fig. 7. Looking down the first column, it can be seen that the maximum of *B* increases and creeps up the red curve as the strength of vaccination decreases. Arguably, the purple curve makes it to its 0-asymptote, at least in the first 3 rows. Clearly, the red curve doesn’t quite get there in the two year prediction shown. The second column shows the effect of the immuninization on the Recovered (Immune) population, *R*. It is seen that only the purple curve in the first row comes close to reaching quasi-equilibrium in 2 Years and only for the first row (Strongest Vaccination Intervention). In the third column, the date of Viral Extinction can be determined from when deaths per day drops below 1, and the results are summarized below in Table 3 below. The fourth column again corresponds to total Deaths. The text annotations at the right indicate total predicted mortality with (red) and without (purple) vaccination of the strength indicated at the far left. Note the lives saved decreases from a factor of ten ($\xi =0.008$) to a factor of two ($\xi =0.002$), emphasizing the importance of vaccination roll-out rates.

**Table 3:** Viral extinction dates and total fatalities as a function of vaccination rapidity, for the scenarios shown in Fig. 7.

$\xi$	$\tau _{\Omega \dot{D}}$	$\tau _{\Omega }$	$D_F$
0.008	785	949	$1.60\times 10^6$
0.006	857	1035	$2.17\times 10^6$
0.004	965	1165	$3.41\times 10^6$
0.002	1135	1379	$6.33\times 10^6$
None	1606	2207	$1.24\times 10^7$

Tables 2 and 3 summarize the predictions of the proposed model on the Duration of the Pandemic. We examine values concerning viral extinction. First, $\tau _{\Omega \dot{D}}$ where $\dot{D} (t) = 0,\ \forall {t>\tau _{\Omega -\dot{D}}}$ indicating that the worst of the pandemic is past, and true viral extinction, $\tau _\Omega$, where $E(t)=I(t)=Q(t)=0, \forall {t>\tau _{\Omega }}$ as defined in above, as well as the total deaths.

Table 2 shows the effect of 5th protective intervention without vaccination and Table 3 shows the effect of vaccination without the 5th intervention. We see here, that for protective interventions the deaths can curbed and the worst of the pandemic can be avoided, but the pandemic must still run its course to achieve true viral extinction which will take multiple years. Moreover, since $P_F$ is high for all the scenarios in Table 2 a further release intervention would result in another spike of the infected population and more deaths. Such a result would be consistent with the pandemic as observed in Europe where extensive social distancing mandates nearly eradicated the virus, but as those mandates laxed new surges were seen nearly everywhere. However, with extreme vaccination implemented immediately $\tau _{\Omega \dot{D}}$ and $\tau _\Omega$ can both be achieved within a year and tens of millions of lives can be saved.

## Discussion

A *timed intervention* extended SEIR model was presented and applied to the United States as a whole. In the discussion of Figs. 4 and 5 it was shown how, by controlling the magnitude of the bilinear term *B*, the *calamitous rapid rise and fall* of predator-prey models can be controlled, and even be actually be eliminated. Thus the desiderata of viral extinction can be realized. However it was also shown that this would require that around half of the total population be placed into lockdown which is unlikely to be feasible in the majority of high population density western first world countries. These results were obtained with the most complete SEIR model extension employed to date, and with a parameter set which produces an almost perfect fit to the deaths per day and total deaths per day in the analyzed date range (see the discussion of Fig. 5).

Our findings show that over a wide range of initial conditions and parameter sets, the presented model correctly predicts model behaviors with multiple qualitatively distinct phases. Also, we have given complete simulation and/or projected waveforms for all 7 of the component populations, whereas other studies focus more narrowly on vital statistics. More critically, we believe that this type of timed intervention model could be used to correlate the effects of real socio-political decisions directly to pandemic measurables like deaths per day. Potentially, the effects of certain protective measures (such as vaccination acceptance or adherence to mask mandates) could be quantified by applying the model to different states (or even counties) in the US with different levels of engagement towards these measures. We believe this model could be used, in conjunction with current economic factors, to recommend a direct intervention schedule which maximizes the number of survivors under realistic socioeconomic conditions.

We have presented extensive treatment of protective interventions of various degrees of timing values and severity. Also we have presented extensive treatment of the effects of vaccination. In this regard we have defined and computed viral extinction. For a representative set of supply dependent vaccination rollouts, we have given final mortality and theoretical final extinction dates. A key finding of our study, is that unless prompt, effective protective interventions, or extensive vaccination interventions are introduced, the pandemic can continue for many years and result in the deaths of many millions in the USA alone. The model is freely available for use and implemented in an easy-to-use iPython Jupyter Lab notebook (link in “Methods” section), meaning that epidemiologists, economists, and politicians can use the model immediately for help with long term predictions during the COVID-19 pandemic. The portion of the pandemic treated in this manuscript, as well as the portion that has occurred since, shows clearly that the progression of a pandemic cannot be modeled effectively by a single set of parameters. The pandemic evolves and changes as a function of time, mutation, political decisions, and social behaviors, and thus, so must the model parameters. The SPEIQRD model proposed here, gives a new tool to predict the rise and fall of the pandemic in large and small populations with time-resolved interventions to deal with these complexities, making it a powerful potential tool to understand and combat both the current and future pandemics.

## Methods

Data for this paper is taken from the New York Times COVID-19 data github (https://raw.githubusercontent.com/nytimes/covid-19-data/master/rolling-averages/us.csv).

Final analyses for the paper were conducted on December 16th, 2020 (corresponding to day 332). The deaths per day data of Fig. 1 are taken direclty from the github source, and the total deaths were determined by integrating the deaths per day values. Data is smoothed by taking a 7-Day rolling average from Day 3 through Day 328.

The odeint function from the python3 *SCIPY* distribution was used to solve these 7 ordinary ordinary differential equations over the closed time interval $t \in [0,T]$, where *t* represents time in days, and *T* is the final time in days. Note that a one year (from January 22) simulation would only extend about 150 days into the future, the current time, *t*, is past the middle of the interval $t\in [0,T]$. We re-iterate the point that, in the simulations of this paper, only unit time steps are taken, as in displayed data from the model. Of course the odeint function takes arbitrarily smaller steps as required to deal with the waveform slope discontinuities frequently encountered in this study. Only the values at the unit times $t=1,2,\ldots ,T$ are recorded. Fortunately, for the interesting part of the parameter space, the solutions of these equations are well-behaved, and have been used frequently as epidemiology models for over a century, including multiple times this millennium. Computationally, this simulation is easy, even trivial. Numerically, however, the simulation can be difficult, involving subtraction of large, almost equal terms, as well as discontinuities in the first derivative of the computed waveforms. For all predictions we use the following initial conditions vector.

**Figure 8:**
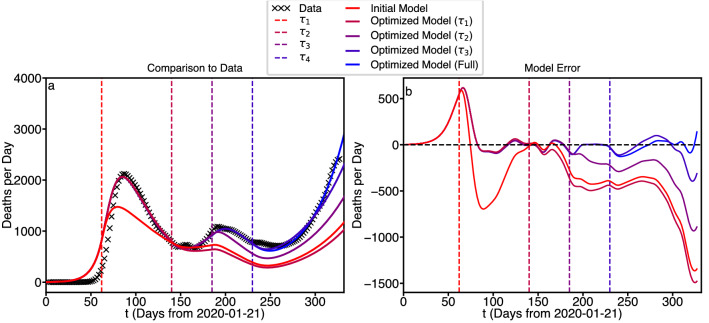
Intervention by intervention optimization of pandemic model.

To model the interventions, we start by selecting intervention values that generate a model with the correct general behavior in terms of deaths per day (i.e., model DPD goes up when data DPD goes up, model DPD goes down when data DPD goes down. The initial state populations are shown in Table 4 and the intervention values are shown in Table 5. Subsequently, we proceed through the model, one intervention at a time, and using the *SCIPY* function minimize to refine the parameters to achieve the best match to deaths per day between the previous intervention and the subsequent intervention. Meaning for $\tau _1$ we fit between $t=0$ and $t=\tau _2$, for $\tau _2$ we fit between $t=\tau _1$ and $t=\tau _3$, for $\tau _3$ we fit between $t=\tau _2$ and $t=\tau _4$, and for $\tau _4$ we fit between $t=\tau _3$ and the end of the data $t=332$). The results are shown in Fig. 8.

It is important to note that this model is under-determined in the sense that there are far more parameters than equations for the optimization. As a result, different $\alpha$ and $\phi$ values than the ones shown can also result in strong fits. This also prevents optimization of the $\tau$ values (which by comparing Table 1 with Table 5 can be seen to be identical) as $\alpha$ and $\phi$ values can be found that minimize error in the model at any given $\tau$. For a more rigorous optimization additional objective functions (other than deaths per day) are needed.

**Table 4:** Initial state populations used for pandemic modeling.

$S_0$	$P_0$	$E_0$	$I_0$	$Q_0$	$R_0$	$D_0$
$N-(P_0+E_0+I_0+Q_0+R_0+D_0)$	100000	4000	50	0	0	0

**Table 5:** Initial intervention parameters used for pandemic model optimization.

$\tau _i$	$\alpha _i$	$\phi _i$
62	0.4	0.03
140	0.1	0.02
185	0.5	0.02
230	0.06	0.02

The model is implemented in an iPython notebook that can be easily altered (to account for different intervention parameters) and used to model and predict the pandemic. It is freely available for download at https://github.com/hachteja/Timed-Interventions.

## Data Availability

All data generated or analyzed during this study are included in this published article.

## References

[1] Kermack WO, McKendrick AG (1927). A contribution to the mathematical theory of epidemics. Proc. R. Soc. Lond. A.

[2] Peng, L., Yang, W., Zhang, D., Zhuge, C. & Hong, L. Epidemic analysis of covid-19 in china by dynamical modeling. arXiv preprint arXiv:2002.06563 (2020).

[3] Carletti T, Fanelli D, Piazza F (2020). Covid-19: The unreasonable effectiveness of simple models. Chaos Solitons Fract. X.

[4] Yamana, T., Pei, S., Kandula, S. & Shaman, J. Projection of COVID-19 cases and deaths in the US as individual states re-open May 4, 2020. Preprint at 10.1101/2020.05.04.20090670 (2020).

[5] Chen Y, Cheng J, Jiang Y, Liu K (2020). A time delay dynamical model for outbreak of 2019-nCoV and the parameter identification. J. Inverse Ill-posed Probl..

[6] Pearl R, Slobodkin L (1976). The growth of populations. Q. Rev. Biol..

[7] Rappole J (2013). The Avian Migrant.

[8] Lofgren ET (2014). Opinion: Mathematical models: A key tool for outbreak response. Proc. Natl. Acad. Sci..

[9] Zhao H, Feng Z (2020). Staggered release policies for COVID-19 control: Costs and benefits of relaxing restrictions by age and risk. Math. Biosci..

[10] Adak D, Majumder A, Bairagi N (2021). Mathematical perspective of COVID-19 pandemic: Disease extinction criteria in deterministic and stochastic models. Chaos Solitons Fract..

[11] Liu X-X, Fong SJ, Dey N, Crespo RG, Herrera-Viedma EA (2021). A new seaird pandemic prediction model with clinical and epidemiological data analysis on COVID-19 outbreak. Appl. Intell..

[12] Hachtel G, Brayton R, Gustavson F (1971). The sparse tableau approach to network analysis and design. IEEE Trans. Circuit Theory.

[13] Anderson RM, May RM (1979). Population biology of infectious diseases: Part I. Nature.

[14] Lorenz EN (1967). The Nature and Theory of the General Circulation of the Atmosphere.

[15] Yi N, Zhang Q, Mao K, Yang D, Li Q (2009). Analysis and control of an SEIR epidemic system with nonlinear transmission rate. Math. Comput. Model..

[16] IHME COVID-19 Forecasting Team (2020). Modeling COVID-19 scenarios for the United States. Nat. Med..

[17] Wong GN (2020). Modeling COVID-19 dynamics in Illinois under nonpharmaceutical interventions. Phys. Rev. X.

[18] Du, Z. *et al.* Covid-19 healthcare demand projections: 22 texas cities. *UT COVID-19 Consortium* (2020).

[19] Cao, L., Liu, Q. & Hou, W. Covid-19 modeling: A review. arXiv preprint arXiv:2104.12556 (2021).

[20] Atkeson, A. A parsimonious behavioral SEIR model of the 2020 COVID epidemic in the United States and the United Kingdom. Tech. Rep., National Bureau of Economic Research (2021).

[21] Friedman J (2021). Predictive performance of international COVID-19 mortality forecasting models. Nat. Commun..

